# Function focused care in hospital among nurses and patients from geriatric and neurology wards: A mixed method process evaluation

**DOI:** 10.1016/j.ijnsa.2025.100401

**Published:** 2025-08-05

**Authors:** Selma Kok, Elke G.E. Mathijssen, Lisette Schoonhoven, Carolien Verstraten, Silke F. Metzelthin, Nienke Bleijenberg, Janneke M. de Man-van Ginkel

**Affiliations:** a Julius Centre for Health Sciences and Primary Care, University Medical Centre Utrecht, Utrecht University, Heidelberglaan 100, 3508 GA Utrecht, the Netherlands; b University of Applied Sciences Utrecht, Heidelberglaan 7, 3584 CS, Utrecht, the Netherlands; c School of Health Sciences, Faculty of Environmental and Life Sciences, University of Southampton, UK; d Diakonessenhuis, Postbus 80250, 3508 TG Utrecht, the Netherlands; e Department of Health Services Research, Care and Public Health Research Institute (CAPHRI), Faculty of Health, Medicine and Life Sciences, Maastricht University, Postbus 616, 6200 MD Maastricht, the Netherlands; f Living Lab in Ageing and Long-term Care, Postbus 616, 6200 MD Maastricht, the Netherlands; g Academic Nursing, Leiden University Medical Centre, PO Box 9600, 2300 RC, Leiden, the Netherlands

**Keywords:** Activities of daily living [MeSH], Function focused care, Functional decline, Hospitals [MeSH], Length of stay [MeSH], Mobility, Nursing staff, Hospital [MeSH], Nursing care [MeSH], Patient-centered care [MeSH], Process evaluation

## Abstract

**Background:**

Function Focused Care is a promising approach stimulating physical activity of patients admitted to hospital. In studying the effectiveness, patients receiving Function Focused Care in Hospital were admitted 3.3 days shorter than patients receiving usual care. However, no differences were found in functional status. Process evaluations alongside an effect studies create an understanding implementation fidelity, of mechanisms of impact, contextual factors.

**Objective:**

to gain insight into the experiences of nurses and patients, and to give insight into implementation fidelity, mechanisms of impact, and contextual factors while implementing Function Focused Care in Hospital on geriatric and neurologic hospital wards.

**Design:**

convergent parallel mixed-methods study, alongside a stepped wedge clinical trial.

**Setting(s):**

four hospital wards of two Dutch hospitals

**Participants:**

nurses, nursing students and care assistants (*n* = 123), and patients (*n* = 24).

**Methods:**

Data for the process evaluation was collected before the implementation of Function Focused Care in Hospital (T0), directly after implementation (T1), and four months after implementation (T2). Data was obtained from patient interviews, focus group interviews with nurses, questionnaires, observations, screening of electronic patients’ records, and logbook notes. For the qualitative data thematic analyses and for quantitative data descriptive statistics were used. The results of these analyses were synthesized into overarching findings.

**Results:**

Findings from the observations showed the care delivery according to the Function Focused Care in Hospital principles increased from 60 % in the control condition to 75 % in the intervention condition. Moreover, nurses stated that patients were only assisted when needed and nurses often asked about their abilities to perform ADL and then encouraged them to do as much as they could themselves. Nevertheless, patients don’t recognize Function Focused Care in Hospital. The results suggest that the implementation fidelity was influenced by different working mechanisms and contextual factors which led to different experiences from nurses, coaches and patients in promoting patient engagement and functional independence. The synthesized findings show that lessons can be learned with regards to continuity of the care provided in the interprofessional collaboration, the challenge in providing personalized care given the current time constraints, the phenomenon of being unconscious incompetent observed in the nurses, and difficulties in demonstrating nursing leadership and autonomy.

**Conclusions:**

Implementation of Function Focused Care in Hospital improved care delivery but highlighted challenges in personalized care, interprofessional collaboration, and nursing leadership, influenced by various contextual factors and working mechanisms.

**Registration:**

https://onderzoekmetmensen.nl/en/trial/24287 (date of first recruitment: 05–02–2016)

**What is already known**

• Implementation of Function Focused Care in Hospital reduces the hospital stay with 3.3 days compared with usual care
• After implementation of Function Focused Care in Hospital, no differences were found in functional status at discharge, three and six months after discharge.
• Process evaluations, alongside effectiveness studies, create insight in implementation fidelity, of working mechanisms, and contextual factors that affect outcomes, potentially influencing the generalizability and practical application of the findings.

Alt-text: Unlabelled box

**What this paper adds**

• Implementation of Function Focused Care in Hospital leads to increased awareness of nurses regarding physical functioning of patient admitted to the hospital.
• While individual nurses know how to work with Function Focused Care in Hospital, there is a need for greater emphasis on leadership and autonomy among nurses to effectively integrate it into the team's workflow.
• When implementing Function Focused Care in Hospital, continued emphasis is needed on the continuity of care provided in the interprofessional collaboration, the challenge in providing personalized care given the current time constraints, the phenomenon of being unconscious incompetent observed in the nurses, and difficulties in demonstrating nursing leadership and autonomy.

Alt-text: Unlabelled box

## 1 Background

Nurses play a pivotal role in supporting patients during a hospital stay toward recovery and restoring their previous functional status (Englebright et al., 2014; Kitson et al., 2013; Kirkevold, 2010). This involves assisting patients with Activities of Daily Living (ADLs) and promoting mobility as an essential component of care (Kitson et al., 2010; Zwakhalen et al., 2018). However, when task completion is prioritized over actively involving patients in their care, this can contribute to a decline in patients’ ADL abilities and functional status, ultimately fostering dependency (Resnick et al., 2012). This tendency towards task-oriented care is reinforced by nurses' inherent desire to attend to their patients, mainly when patients are vulnerable (Resnick et al., 2012). Consequently, there is a pressing need for a paradigm shift in nursing care toward strategies to prevent, maintain, or enhance patients' functional capabilities (Loyd et al., 2020).

Multidisciplinary interventions like 'reablement' and 'Function Focused Care' have emerged as promising strategies, emphasizing a shift from passive caregiving to active patient participation to strengthen patients’ functional capabilities (Kok et al., 2024; Vluggen et al., 2024). Function Focused Care is defined as "…a philosophy of nursing care that focuses on evaluating the older adult's underlying capability with regard to function and physical activity and helping him or her optimize and maintain functional abilities and increase time spent in physical activity…” (Resnick et al., 2012, page 4). This approach underscores the importance of evaluating patients' functional capabilities and customizing care to promote sustained physical activity and participation in all aspects of daily living (Galik et al., 2013; Resnick et al., 2013).

Function Focused Care has demonstrated effectiveness in optimizing and preserving function while increasing engagement in physical activities within hospitals in the United States (Lee et al., 2019; Boltz et al., 2015, 2023). To explore its potential in a different context, Function Focused Care was adapted and transferred from its established use in the United States to the Dutch hospital setting, with adjustments made to align with local healthcare practices and patient populations. Following the Medical Research Council (MRC) framework for complex interventions (Craig et al., 2008), we studied the applicability of Function Focused Care in the Dutch hospital setting in a feasibility study (Kok et al., 2021). The effectiveness of Function Focused Care in Hospital was evaluated in neurological and geriatric patients acutely admitted to hospital in cluster randomized stepped-wedge trial (Kok et al., 2024). In the effect study, an average decrease in hospital admission by 3.3 days for the Function Focused Care in Hospital condition was observed. Patients were also discharged to their home more often when receiving Function Focused Care in Hospital (Kok et al., 2024).

However, conducting effect studies without process evaluations creates a gap in understanding and explaining the mechanisms of impact, contextual factors, and implementation fidelity. Therefore, alongside the effect study, a process evaluation was conducted. This process evaluation aims to gain insight into the experiences of nurses and patients, and to give insight into implementation fidelity, mechanisms of impact, and contextual factors while implementing Function Focused Care in Hospital on geriatric and neurologic hospital wards. With this knowledge, we aim to explain the methodology and enhance effectiveness by optimizing the implementation of Function Focused Care in Hospital for different healthcare systems, thereby improving patient outcomes and optimizing hospital stay duration in a more context-specific manner**.**

## 2 Methods

### 2.1 Design

We conducted a process evaluation using a convergent mixed-methods design, alongside the effect study. Both qualitative and quantitative data were collected and analysed separately, and then integrated during the interpretation phase to provide a comprehensive understanding of the implementation process. This design allowed us to explore not only the outcomes but also the contextual and experiential dimensions of function-focused care (Creswell and Piano Clark, 2007). We used the process evaluation framework of the Medical Research Council as a theoretical base for this study (Moore, 2015). This framework provided a theoretical foundation for examining the implementation process, mechanisms of impact, and contextual factors. We collected data over 18 months between February 2016 and March 2018 in both the control condition (usual care) and intervention condition (Function Focused Care in Hospital). Initially, all participating wards served as the control condition and, one by one transitioned into the intervention condition via a transition period (Kok et al., 2024).

### 2.2 Setting and participants

This study was conducted on the neurological and geriatric wards of two Dutch hospitals, one university medical center and one general hospital, both located in the middle of the Netherlands. The study population comprised nurses and patients, who were selected through a convenience sampling method. To be included in the study, nurses needed to work at one of the four participating wards. All nurses of the participating wards were asked to participate. Patients of these wards were included if they were aged ≥18 years and admitted to one of the participating wards with a recent (onset ≤ 1 week) stroke (neurological wards) or who were treated by the geriatrician (geriatric wards). Patients were included regardless of their ability to communicate adequately due to cognitive decline or aphasia to obtain a representative sample of the population. Patients were excluded if they were expected to stay in the hospital <48 h, had a life-threatening condition, were terminally ill, did not master the Dutch language, or had been previously enrolled in this study.

### 2.3 Intervention

In the intervention condition, all patients received care according to the Function Focused Care in Hospital approach. A detailed description of this approach following the Template for Intervention Description and Replication (TIDieR) has been previously published (Kok et al., 2021). Function Focused Care in Hospital concerns a) patient and nurse interaction, b) involvement of patients' families and the multidisciplinary team, and c) evaluation of ward environment, policies and culture (Resnick et al., 2012). These aspects are affected by four key components: 1) Environmental and Policy Assessment: Evaluating ward policies, cultural norms, and environmental factors. 2) Education: Training nurses to educate patients about their role in movement during the hospital admission, inform their families, and collaborate with the multidisciplinary team. 3) Goal Setting with the Patient: Emphasizing collaborative goal setting based on the patient’s physical and psychological capabilities and preferences, involving both long-term and daily goals. 4) Ongoing Motivation and Mentoring: Training for nurses to motivate patients and their families while enhancing their self-assurance, in close collaboration with the multidisciplinary team.

The intended stakeholders of Function Focused Care in Hospital include nurses, patients, patients' families, and the multidisciplinary team.

### 2.4 Usual care

In the usual care condition, patients received standard practices. Within Dutch hospital settings, usual care primarily focuses on patient recovery and is administered by a multidisciplinary team of physicians, nurses, nurse assistants, physiotherapists, and other healthcare professionals. While goal setting and activity plans are outlined in the nursing care, their implementation varies. Patient involvement in goal setting is infrequent, and there is no established policy for family participation in nursing care. Consequently, patient and family involvement largely depends on the individual preferences.

### 2.5 Implementation

During the four-month transition from the control to intervention, implementing Function Focused Care in Hospital involved comprehensive nurse training. This allowed nurses to familiarize themselves with the approach. The researcher (CV) introduces the four components of Function Focused Care in Hospital and their application in daily nursing practice. A two-hour training session emphasized the benefits of physical activity for patients. Designated coaches would encourage colleagues to integrate Function Focused Care in Hospital into their daily routines. Written materials were distributed to patients' families and the multidisciplinary team. A thorough description of the intervention was previously documented in the feasibility study (Kok et al., 2021). The transition phase was excluded from both conditions, so no data was collected during this period.

### 2.6 Procedures of data collection

Qualitative data was collected by observing and interviewing participants and quantitative data was collected by patient file screening and questionnaires. All outcomes were collected by trained research assistants or the researcher (CV). Table 1 presents an overview of the data collection.

**Table 1:** Data collection and timepoints.

	Before implementation (T0)	Directly after implementation (T1)	4 Months after implementation (T2)
**Qualitative data sources**			
Patient interviews		X	X
Focus group interviews nurses	X	X	X
Focus group interview coaches			X
Observations	X	X	X
**Quantitative data sources**			
File screening	X		X
Questionnaires			
Evaluation training		X	
Barriers Facilitators Assessment Instrument	X	X	X
Nurses Outcome Expectations	X	X	X
Self-Efficacy Nurses	X	X	X
Culture Environment	X	X	X
Knowledge Test	X	X	X
Team Learning Assessment Score	X		

#### 2.6.1 Qualitative data

Researchers (CV, JMG, and SV) collected the qualitative data through focus group interviews with nurses, individual interviews with patients, and observations of participants during ADL or mobilization care moments at the participating wards.

The patient interviews were open, focusing on patients’ experiences about some of the core elements of Function Focused Care in Hospital: determining long- and short-term goals with the nurse, visibility of the goals in the patients' hospital rooms, stimulation to actively engage in daily care activities, stimulation to get out of bed as much as possible, and information patients received about being physically active. Individual interviews with patients were conducted at T1 and T2.

Topics that guided all the focus group interviews on the different time points (T0, T1, and T2) were experiences with physical activity during daily care, the experienced workload, the implementation of Function Focused Care in Hospital, and the experienced barriers and facilitators. In the focus group interview with the coaches, we also asked about their experiences regarding being a coach on the ward. Focus group interviews with nurses were conducted at the start of the control condition (T0), directly after the implementation (T1) and 4 months after the implementation (T2).

The observations were conducted during care activities (ADL/mobility) and focused on observing whether care was delivered according to Function Focused Care in Hospital. The outcomes that were measured were the number of activities observed, the number of activities applied according to Function Focused Care in Hospital, and the percentage of care moments according to Function Focused Care in Hospital. Observations were conducted at T0, T1, and T2.

During the study, the researcher (CV) kept a logbook with details about the implementation per ward, such as policy changes, who sent invitations, the duration of the education, the coaches' experiences, etc.

#### 2.6.2 Quantitative data

Trained research assistants or researchers not involved in the data analyses collected the quantitative data. The patient files were screened to determine what was reported regarding washing, dressing, transfer, and mobilization, including the percentage of reported goal setting, and the content of the goals (T0, T1, and T2). An attendance list was maintained for the initial training of the nurses.

Furthermore, nurses completed different questionnaires at T0, T1 and T2. They were asked to fill in the Barriers and Facilitators Assessment of Implementation (BFAI) to give insight into the experienced barriers and facilitators regarding the implementation of Function Focused Care in Hospital (Peters M.A.J. et al., 2003). The BFAI contains 27 statements about possible barriers to Function Focused Care in Hospital, rated with a 5-point Likert scale from 1 (fully disagree) to 5 (fully agree). The total score can range from 27 to 137, with 137 as the highest score representing the lowest experienced or expected barriers regarding Function Focused Care in Hospital by nurses.

The Nurses Outcomes Expectations (NOE) was filled in to give insight into the nurses’ expectations for the outcomes of Function Focused Care in Hospital (Resnick and Simpson, 2003). The NOE contains nine statements regarding the possible benefits of Function Focused Care in Hospital, with a Likert scale of 1 (fully disagree) to 5 (fully agree). The total score can range from 9 to 45, with 45 as the highest outcome expectation regarding Function Focused Care in Hospital.

The Self Efficacy Nurses (SEN) was used to gain insight into the self-efficacy in applying Function Focused Care in Hospital in daily practice (Resnick and Simpson, 2003). The SEN contains ten questions with a 5-point Likert scale from 1 (no trust) to 5 (fully trust). The total score can range from 10 to 50, with 50 as the highest self-efficacy regarding Function Focused Care in Hospital.

The Team Learning Assessment Score (TLAS) gives insight in the experienced team learning on the ward (van Offenbeek, 2001). The TLAS contains 26 statements, 16 relating to how your team handles knowledge and information relating to your work, and 10 relating to the communication within the team at the ward level. All statements were rated with a 5-point Likert scale, 1 (fully disagree) to 5 (fully agree). The total score can range from 26 to 130, with 130 as the best possible team learning to implement Function Focused Care in Hospital.

The experienced culture and environment (CE) of the ward regarding Function Focused Care in Hospital was questioned by 12 statements with a 5-point Likert scale from 1 (fully disagree) to 5 (fully agree). The total score can range from 12 to 60, with 60 as the best possible culture and environment to implement Function Focused Care in Hospital.

The nurses' knowledge was tested using a Knowledge Test (KT) with 13 multiple-choice questions about Function Focused Care in Hospital.

The BFAI, NOE, SEN, CE, and KT were collected at T0, T1 and T2, and the TLAS was collected at T0. All questionnaires were translated into Dutch, using a forward backward translation procedure with bilingual Dutch and English native speakers, before they were sent to the nurses via SurveyMonkey.

The training was evaluated using 12 questions about the nurses' experiences with regards to the training (T1).

#### 2.6.3 Baseline

The baseline characteristics of the nurses, including age, years of experience working at the participating ward, profession, and educational level, were collected through questions at the start of the focus group interviews. Additionally, data on patients' age and length of stay were collected at the start of the individual interviews.

### 2.7 Data analysis

#### 2.7.1 Baseline characteristics

The baseline characteristics are presented with descriptive statistics such as mean and Standard Deviation (SD) for continuous normally distributed variables and with a median and Inter Quartile Range (IQR) for continuous not normally distributed variables.

#### 2.7.2 Qualitative data analysis

We used inductive qualitative analysis methods for analyzing the data from the focus group interviews with nurses and patient interviews. First, we separated the transcripts per timepoint: T0, T1, and T2. We started with identifying meaningful segments in the transcripts, to which we assigned initial in-vivo codes. These codes were then grouped into categories based on shared meaning. This was done independently by the researchers (SK, EM). The categories were described and listed for each time point (T0, T1, and T2), allowing us to examine similarities and differences over time. Furthermore, we discussed relevance based on the research questions. In a meeting with a third researcher (JMG) we reached consensus about the relevant findings. Qualitative data analysis was supported by NVivo (version 12).

#### 2.7.3 Quantitative data analysis

Quantitative data were presented using descriptive statistics such as mean and SD for continuous, normally distributed variables and with a median and IQR for continuous, not normally distributed variables. Categorial variables were analyzed and presented with numbers and percentages.

We calculated a total score, group means, and SD per timepoint for all the questionnaires (BFAI, NOE, SEN, TLAS, CE, and KT). To test for differences between time points, we compared group means over time with mixed effects models. Mixed effects models were suitable for dealing with the missing data that occurred.

#### 2.7.4 Data syntheses

We synthesized the qualitative and quantitative data (SK, EM) by integrating the datasets based on common themes, shared research questions and emerging themes. Using thematic analysis, we identified key themes that emerged from both data types. We then examined the degree of convergence (where findings aligned) or divergence (where findings offered complementary or differing perspectives). This triangulation approach (SK, EM, JMG) enhanced the validity of our interpretations. Direct quotes from both the focus group and individual interviews were included to enrich the findings and provide deeper insights. The synthesized data and themes were systematically presented, highlighting the integration of qualitative and quantitative insights. Quotes from the interviews were used to support the findings.

### 2.8 Informed consent and ethics approval

The study was exempted from Medical Research Involving Human Subjects Act by the Medical Ethics Board of the University Medical Centre of Utrecht (protocol number 15/517).

The ward managers consented to participate after they had received verbal and written information regarding the intervention and study. All nurses working at one of these wards were approached during regular team meetings and via email and gave informed consent for participation in the data collection during the different implementation activities: education sessions, bedside teaching and focus group interviews. Patients who gave informed consent to participate in the parallel effect study were asked for agreement to be invited for individual interviews for this process evaluation.

The study was registered on https://onderzoekmetmensen.nl/en/trial/24287 (date of first recruitment: 05–02–2016). Registration occurred after recruitment commenced, but before the end of the trial. The trial was conducted in accordance with the protocol that was approved for funding and reviewed by the ethics committee prior to first recruitment. The registration reflects this protocol. Documentation is available from the author on request.

## 3 Results

### 3.1 Baseline characteristics

During this process evaluation, 167 care professionals worked at the four wards: 128 nurses, 28 nursing students, 7 care assistants, and 4 other care professionals. The median age of the care professionals was 31.5 years (IQR 23.8 – 47.3), the mean years of working at the ward were 7.6 (SD 8.1), and the median years they had a degree for the profession they worked in was 7.0 (IQR 2 – 18.5). The response rate of the questionnaires for the care professionals differed per timepoint and per questionnaire between *n* = 35 (21.0 %) and *n* = 98 (58.7 %).

In total, 46 care professionals participated in a focus group interview: 37 nurses, 4 nurse assistants, and 5 nursing students. Eleven nurses participated in more than one focus group interview, meaning that they participated at different time points or in a focus group with nurses and a focus group with coaches. The mean age of nurses, nurse assistants, and students who participated in a focus group interview was 37 years (SD 12.0), and the mean years working at the ward was 7.4 (SD 5.9). Patients (*n* = 24) who were interviewed had a mean age of 72 years (SD 12.9) and were admitted to the hospital for an average of 10 days (SD 4.3). Eleven patients were admitted to one of the neurology wards, 13 patients were admitted to one of the geriatric wards. Baseline characteristics are presented in Table 2.

**Table 2:** Baseline measurements.

**Nurses, nursing students and care assistants (***n* = 160)	
Age (median (IQR))	31.5 (23.8–47.3)
Years degree of the profession they work in (median (IQR))	7.0 (2.0–18.5)
Years working on the ward (mean (sd))	7.6 (8.1)
Profession (n (%))	
Nurse (n (%))	123 (76.9)
Nursing student (n (%))	28 (17.5)
Care assistant (n (%))	7 (4.4)
Other (n (%))	2 (1.3)
Attendance (n (%))	
Total attendance training	84 (47.7)
Total attendance bedside teaching, lunch meetings, and/or clinical classes	97 (55.1)
Nurses (*n* = 123)	
Attended training (n (%))	70 (56.9)
Attended bedside teaching, lunch meetings, and/or clinical classes (n (%))	72 (58.5)
Nursing students (*n*= 28)	
Attended training (n (%))	7 (25)
Attended bedside teaching, lunch meetings, and/or clinical classes (n (%))	19 (67.9)
Care assistants (*n*= 7)	
Attended training (n (%))	7 (100)
Attended bedside teaching, lunch meetings, and/or clinical classes (n (%))	6 (85.7)
**Patients**	
Included patients for interview	(*n*= 24)
Age (median (IQR))	79 (62 - 83)
Length of stay (median (IQR))	10 (5 – 12)

### 3.2 Findings

Details regarding quantitative and qualitative findings about experiences of nurses and patients with Function Focused Care in Hospital, described in terms of implementation fidelity, mechanism of impact, and contextual factors, are presented in Table 3, Table 4, respectively. In Table 5, the results of the mixed effects models for the questionnaires are presented.

**Table 3:** Quantitative findings.

	Before implementation (T0)	Directly after implementation (T1)	Four months after implementation (T2)
**Observations during ADL care**	(*n* = 31)	(*n* = 9)	(*n* = 32)
Percentage Function Focused Care in Hospital applied (median [IQR])	60.0 [41.43–87.50]	83.33 [82.50, 100.00]	75.00 [57.14, 89.29]
Short term goal during observation (n (percentage))	0 (0)	2 (16.7)	1 (3.1)
**File screening**			
**Goal setting** (n (%))			(*n* = 39)
At least one short term goal in patient file (n (%))			11 (28.2)
Short term goal evaluated one time in patient file (n%))			3 (7.7)
Long term goal in patient file (n (%))			10 (25.6)
**Nursing reports topics**	(*n* = 449)		(*n* = 390)
Bathing (n (%))			
Described what patient/nurse did	33 (7.4)		26 (6.6)
Not described what patient/nurse did and patient in need of help	69 (15.4)		69 (17.7)
Patient is independent	18 (4.0)		47 (12.1)
Not reported in patient file	321 (71.5)		242 (62.1)
Missing in dataset	8 (1.8)		6 (1.5)
Dressing (n (%))			
Described what patient/nurse did	6 (1.3)		8 (2.1)
Not described what patient/nurse did and completely dependent patient	70 (15.6)		70 (17.9)
Patient is independent	12 (2.7)		41 (10.5)
Not reported in patient file	353 (78.6)		265 (67.9)
Missing in dataset	8 (1.8)		6 (1.5)
Transfer Dependency (n (%))			
Not dependent	24 (5.3)		14 (3.6)
Partly dependent	67 (14.9)		63 (16.2)
Completely dependent	23 (5.1)		18 (4.6)
Not reported in patient file	327 (72.8)		292 (74.8)
Missing in data set	8 (1.8)		3 (0.8)
Mobility Dependency (n (%))			
Not dependent	49 (10.9)		39 (10.0)
Partly dependent	95 (21.2)		117 (30.0)
Completely dependent	1 (0.2)		2 (0.5)
Not reported in patient file	296 (65.9)		229 (58.8)
Missing in dataset	8 (1.8)		3 (0.8)
**Evaluation training nurses (*n*=31)**			
Content (median [IQR])		8.0 [7.0, 8.0]	
Form (median [IQR])		8.0 [7.0, 8.0]	
Teacher (median [IQR])		8.0 [8.0, 8.0]	
**Questionnaires nurses (mean sum score (sd))**			
Barriers and Facilitators Assessment of Implementation	78.4 (13.9) (*n* = 43)	86.3 (11.1) (*n* = 68)	80.9 (21.2) (*n* = 40)
Nurses Outcomes Expectations	36.5 (5.1) (*n* = 39)	38.6 (4.1) (*n* = 65)	38.1 (5.7) (*n* = 35)
Self Efficacy Nurses	18.3 (16.4) (*n* = 82)	25.4 (16.3) (*n* = 98)	17.8 (16.9) (*n* = 77)
Team learning assessment Score	81.6 (15.3) (*n* = 38)		
Knowledge Test	5.3 (2.9) (*n* = 53)	6.0 (2.4) (*n* = 74)	5.1 (3.1) (*n* = 51)
Culture and Environment	39.5 (5.6) (*n* = 36)	40.2 (5.7) (*n* = 62)	41.1 (5.0) (*n* = 34)

**Table 4:** Research questions and qualitative findings.

**Data source**		**Logbook**	**Focus group interview**				**Individual interview**	
		Researcher T0-T2	Nurses T0	Nurses T1	Nurses T2	Coaches T2	Patients T1	Patients T2
**Concepts**	**Findings**							
**Implementation fidelity**								
Achievement of delivery (how)	Policy changes included adding a heading for FFC reporting, setting a fixed place for reports, and using whiteboards within patients’ reach for short-term goals.	X						
	The training was provided in two or three sessions, all nurses were invited by the team leader of the ward.	X						
	3 to 5 coaches per ward.	X						
Extent of protocol performance (Fidelity)	Nurses increasingly encouraged patients to perform ADL independently, whereas previously they had performed these activities for patients even though patients could have done them themselves.			X	X	X	X	X
	Goals for physical activity were not typically set collaboratively with patients, which patients felt would have been beneficial.						X	X
	Many patients spent most of the day in bed during hospitalization, attributing this mainly to their deteriorated medical condition.						X	X
	Achieving sufficient physically activity levels was always a focus for patients, even at home.						X	X
	Coaches were able to adapt their role and actions to the ward’s needs, which leaded to differences in the role as coach.	X						
	The multidisciplinary team was informed by one of the coaches or the team leader via email or the study information letter.	X						
Implementation of FFCiH* according to its core principles & adaptions made during the study (dose)	Goal setting was inconsistently implemented. The follow-up and evaluation of goals remain limited due to insufficient record-keeping, with little reporting on progress after goals are initially set. While reminders and requirements prompt goal-setting activities, it is not done voluntarily.			X	X	X		
	Nurses highlight the positive impact of goal setting on patients, who begin to reflect on their own aspirations, but stress the need for improved consistency in recording and tracking goals.			X	X			
	Most goals focus on mobility, with fewer addressing ADLs. Goals are often abstract, making them difficult to translate into practical, achievable objectives.					X		
	A lack of continuity in care and a clear point of contact was a concern.						X	X
	No adaptions to the intervention were made during this study	X						
Reach of the target group	New colleagues were insufficiently introduces regarding Function Focused Care in Hospital.				X			
	Variability among nurses in how much they adopt Function Focused Care in Hospital, with some still taking over tasks from patients despite recognizing the benefits of fostering patient autonomy.					X		
	Patients did not recognized the principles of Function Focused Care in Hospital.						X	X
	The teams required constant motivation, and there were only few proactive questions about Function Focused Care in Hospital from the nursing staff					X		
	Patients primarily referred to physical therapists as the discipline that supports patients’ physical activity during hospitalization. Concrete activities provided as examples were training patients’ mobility and monitoring progress.						X	X
	Patients had varying perspectives on whether it is up to nurses to support patients’ physical activity during hospitalization. Some patients thought that this was not within the nurses’ roles and responsibilities, while others expected a proactive approach from nurses in this regard.						X	X
	Other disciplines were physicians, discussing long-term expectations and preferences for physical activity with patients, and volunteers, assisting patients with exercises.						X	X
	Occasional discussions about goals between patients and nurses, along with some examples where nurses provided help with exercises, offered advice, monitored movement, and discussed the importance of physical activity.						X	X
**Mechanism of impact**								
Change produced by the intervention	Nurses perceived Function Focused Care in Hospital as time consuming.		X					
	Nurses indicated that often they did not prioritize Function Focused Care in Hospital compared to other tasks.			X				
	The implementation efforts focus on aligning practices and addressing barriers to Function Focused Care in Hospital adoption. Nurses discuss points of resistance and identify useful reminders during the implementation process to facilitate Function Focused Care in Hospital integration.			X				
	Nurses became aware that Function Focused Care in Hospital yields time in the long term, even though they did not directly perceive this themselves.				X			
	Nurses indicated that working according to the principles of Function Focused Care in Hospital fits with their profession. Function Focused Care in Hospital aligns with the nurses’ vision of care and fits well with their existing work practices, such as rehabilitative care. They find that with more frequent use, Function Focused Care in Hospital becomes easier to integrate, though reminders from coaches or supervisors are needed to keep it from fading into the background.				X			
	Nurses indicated that they valued Function Focused Care in Hospital for its fit with person-centered care, enabling patients to have a say in their own care.				X			
	Feeling in control during hospitalization was important for patients. The extent to which they felt in control during hospitalization varied from patient to patient.						X	X
	Some patients mentioned that they would have appreciated if nurses had been more active in setting goals, while others felt it unnecessary, stating that they already set goals themselves.						X	X
	Patients believed physical activity to be important for maintaining independence, a belief they held even before hospitalization. They had often heard about the positive effects of physical activity from the media.						X	X
	Four months after implementation, nurses emphasize the importance of not taking over patient activities, despite time constraints.				X			
	Nurses’ preferences regarding continuing working according to the principles of Function Focused Care in Hospital in the future varied. Generally, they preferred to continue with aspects they had experienced positively, such as delegating activities to patients and relatives, while they preferred to discontinue with aspects they had experienced negatively, such as the reporting of goals.				X			
	Differences between nurses in their engagement with Function Focused Care in Hospital. They experienced resistance among their fellow nurses in the early stages of implementation.					X		
Perceived barriers regarding FFCiH	Initial resistance from nurses, particularly due to feelings of exclusion from the process.					X		
	Some nurses mentioned that when they experience a higher workload, they have the tendency to take things over from patients without question.		X	X	X			
	Concerns about the implementation of new practices, particularly when these require additional time or tasks.		X	X				
	A lack of continuity makes the implementation of Function Focused Care in Hospital and the maintenance of patient goals more vulnerable to oversight.			X	X			
	The workload is overwhelming, and having to guide patients in self-care activities, such as washing themselves, consumes additional time nurses feel they do not have.		X			X		
	Mixed experiences with the time Function Focused Care in Hospital demands, often noting uncertainty about whether it ultimately saves time.			X		X		
	An increasing administrative burden, which nurses feel puts the core of their profession under strain. Function Focused Care in Hospital feels like an increase in the administrative burden.		X	X		X		
	Nurses are bound by strict schedules and an ongoing pressure with multiple tasks requiring their attention. This further limits the time available and forces nurses in to prioritizing tasks such as applying Function Focused Care. Function Focused Care in Hospital does not always take precedence.			X	X			
	The competing demands in direct care, often leads nurses struggle to implement Function Focused Care in Hospital and often leads to take over tasks from the patients during busy periods.			X	X			
	No one assumed responsibility for the implementation of Function Focused Care in Hospital and ensuring its ongoing prioritization.				X			
	A lack of understanding of the methods and goals of Function Focused Care in Hospital.					X		
	The manual was seen as impractical.					X		
	Existing routines, such as the expectation that all patients must be washed before coffee time reinforce the time issue.					X		
	Coaches recognize that they could have been more proactive in their role, suggesting that greater initiative on their part might have further supported the integration of Function Focused Care in Hospital into daily practice.					X		
	Physicians were engaged in supporting patient mobility, but not always according to Function Focused Care in Hospital principles.					X		
	Some initial resistance among physiotherapists and occupational therapists, who feared that Function Focused Care in Hospital might interfere with their roles. Over time, there was a desire for better involvement of these disciplines.					X		
	Nurses appeared busy and that they frequently had to wait for assistance.						X	X
	Patients expected a more proactive role from nurses, recognizing the significant impact that nurses can have on their engagement in physical activity.						X	X
	Perceptions of independence in ADL and mobility were mixed, with some patients comparing their current abilities unfavorably to their levels of independence at home.						X	X
	Patients view physiotherapists as the primary providers for movement-related care. They believe that physiotherapists are responsible for stimulating movement, setting goals, and exercising with patients, reinforcing their role as key facilitators in achieving physical activity and mobility goals.						X	X
Perceived facilitators regarding FFCiH	Nurses stress the importance of clear communication regarding the benefits of such changes and note that initiatives originating from within the team encounter less resistance than those imposed externally.		X					
	Nurses display a generally positive attitude towards care related to mobility, they emphasize patient-centered care as a source of satisfaction.		X					
	Nurses display a generally positive attitude towards care related to mobility, they emphasize patient-centered care as a source of satisfaction.		X					
	Nurses also acknowledge some positive aspects of administrative tools, such as checklists, which they find helpful in providing structured insights and guiding care delivery effectively.		X	X	X			
	Some nurses acknowledge that Function Focused Care in Hospital may offer long-term benefits, particularly for the patients.				X			
	The potential for enhancing continuity of care and patient motivation with goal setting.			X	X			
	Emphasis was placed on the importance of nurses providing relatives with clear explanations of their actions and rationale to prevent misunderstandings.		X					
	Nurses consistently expressed a positive attitude towards involvement of relatives in Function Focused Care in Hospital.			X	X			
	Nurses view Function Focused Care in Hospital as patient-centered care, placing control in the hands of the patient while adapting goals and methods to each individual’s capabilities.				X			
	Sharing successes and having a designated person who regularly brings it to attention would contribute to focus on Function Focused Care in Hospital.				X			
	Nurses say that they aim to align care with the patient’s physical abilities, helping patients become more aware and regain control as part of the rehabilitation process.			X				
	Training new staff and continued emphasis on Function Focused Care in Hospital’s value are seen as important for long-term success.			X	X	X		
	Gaining support from the nursing staff to ensure successful implementation.					X		
	Continuous reinforcement of Function Focused Cate in Hospital to prevent it from being overlooked in daily care.					X		
	Sharing examples of success has proven to be helpful.					X		
	Bedside teaching was particularly well-received.					X		
	Patients increasingly emphasized their own initiative and desire to maintain autonomy in their care.							X
	Knowledge about the importance of exercise and staying healthy was largely derived from media or hearsay, and hospital admission did not seem to change these perceptions.						X	X
	Patients consider exercise important for their health.						X	X
**Contextual factors**								
Implementation and outcomes affected by context	A lack of time to the implement Function Focused Care in Hospital.		X	X	X			
	A high workload was experienced. There were many tasks demanding nurses’ attention. Nurses mentioned that there was little room for them to support patients’ physical activity during hospitalization, referring to the tight schedules they had to adhere to during their shifts.		X	X	X			
	Nurses held a general belief that every patient should be washed before coffee time (i.e., 10:00 AM), imposing additional pressure on themselves.		X	X	X			
	A lack of continuity in the workplace made it difficult for nurses to effectively monitor patients’ physical activity, highlighting the vulnerability of oral and written handover between colleagues.			X	X			
	A lack of continuity due to a high staff turnover.					X		
	Nurses were under pressure. Consequently, many patients stated that they took initiatives for exercising on their own, perceiving that nurses were too occupied to assist them with this.						X	X
	Little continuity among nurses was perceived. Patients missed having a consistent point of contact.						X	
	The hospital environment was not always conducive to physical activity. Some patients mentioned that the limited space of the ward they were admitted to restricted physical activity, as they could only walk one circuit, repeatedly.						X	X
	The hospital environment was unfamiliar, which discouraged patients from being more physically active. Patients often did not know where everything was located and environmental adaptations they had at home, such as a handrail, were missing in the hospital.						X	X
	Patients indicated that they were more physically active at home than in the hospital.						X	X
	Patients held a general belief that, during hospitalization, they were confined to the role of patient and nurses were expected to treat them accordingly.						X	X
	Patients’ role at home is different, being part of a household and society. Consequently, different expectations are placed upon them, such as performing domestic tasks, that naturally involve more physical activity.						X	X

*FFCiH = Function Focused Care in Hospital.

**Table 5:** Mean intervention effects for questionnaires for nurses.

	Β Δ before implementation - after implementation	95 % CI	p-value
Directly after implementation			
Barriers and Facilitators Assessment of Implementation	8.04	2.58 – 13.49	0.01
Nurses Outcomes Expectations	2.17	0.36 – 3.97	0.02
Self Efficacy Nurses	6.44	2.52 – 10.37	0.00
Knowledge Test	0.74	−0.14 – 0.83	0.10
Culture and Environment	1.11	−0.87 – 3.10	0.26
Four months after implementation			
Barriers and Facilitators Assessment of Implementation	2.16	−4.05 – 8.35	0.49
Nurses Outcomes Expectations	1.33	−0.77 – −3.43	0.21
Self Efficacy Nurses	0.15	−3.98 – 4.28	0.94
Knowledge Test	−0.12	−1.08 – 0.83	0.80
Culture and Environment	1.35	−0.94 – 3.64	0.24

Based on the syntheses of these quantitative and qualitative data, we identified four themes. 1) Continuity of care was under pressure; nurses experienced a lack of continuity of care due to high staff turnover, work load strains, increased administrative burden, and inconsistencies in care approaches. 2) Being unconscious incompetent: although nurses said they increasingly applied Function Focused Care in Hospital, patients did not recognized core principles of Function Focused Care in Hospital and the file screening showed that reports lack topics such as washing, dressing & mobility. 3) Need for nursing leadership and autonomy: nurses recognized the importance of Function Focused Care in Hospital, but experienced difficulties in showing autonomy and nursing leadership to prioritize the tasks they find important. And 4) Added value to personalized care experiences: nurses preferred delivering personalized care and saw Function Focused Care in Hospital as an addition in providing personalized care where patients’ autonomy is strengthened.

#### 3.2.1 Continuity of care is under pressure

Nurses experienced a lack of continuity of care, which they said was caused by a lack of time, a high workload and a high staff turnover. Nurses perceived time as the most significant barrier to implementing Function Focused Care in Hospital. Workload strains stemming from staff shortages and increasing administrative demands forced the nurses to prioritize multiple competing tasks, often at the expense of engaging patients in their care.

“You just got started and then there is the neuro surgeon, who wants to walk their doctor’s visit […] Gone. And then you’re back, there is the speech therapist, this goes on and on, on a day […] You get pulled away from work very often.” (Quote #1, Neurology nurse, T1).

This behavior was reinforced by the nurses’ belief that Function Focused Care in Hospital is time-consuming; it often seems quicker for the nurses to perform ADL tasks for patients rather than guide them in doing these tasks themselves. Additionally, nurses often hold certain beliefs about what constitutes quality care and the expectations of a competent nurse, for example, ensuring that all patients are washed before coffee time (10 AM). Nurses said that these beliefs could have led them to adopt a task-oriented approach to manage their partly self-imposed workload pressures, resulting in nurses taking over tasks that patients can perform, thereby detracting from the delivery of Function Focused Care in Hospital. Furthermore, nurses stated that administering and evaluating physical activity goals requires time, compounding the pressure on the nurses. Although nurses knew that Function Focused Care in Hospital can save time in the long term, they felt that they needed to experience these benefits in their daily routines.

“And that also applies in the evening shift, if I’m very busy, (…) I don’t have the time to just sit quietly for half an hour and watch how that man – (…) Then I really take over, whereas if I had the time, I would just do that and sit next to him; hey do it yourself. I’ll sit down, try it. I do think that’s unfortunate, I think there is a lot more to be got out of it than can be get out of it now.” (Quote #2, Geriatric nurse, T1)

Nurses also said that a lack of continuity in the nursing team due to high staff turnover exacerbates workload strains. They described that when different nurses care for the same patients, inconsistencies in care approaches arise, including in applying Function Focused Care in Hospital. Nurses acknowledged the potential for enhancing continuity of care and patient motivation and highlight the positive impact of goal setting on patients. Nevertheless, nurses often did not attend to the same patient on consecutive days, which limited their ability to monitor a patient's physical activity during hospitalization effectively. They stressed the need for improved consistency in recording and tracking goals as they must rely on colleague handovers.

“And if you do work and work on it intensively yourself and it doesn’t gets picked up by colleagues, so it falls into a hole every time, then it can be demotivating.” (Quote #3, Neurology nurse, T2)

This created information gaps and disrupted care continuity. Patients also recognized the nurses' need for continuity of care and missed a stable contact person among the nursing staff.

“No point of contact, people are constantly running back and forth. One who comes to draw blood, and one who comes to do that […] if I ask them a question; yes, I want to do this first, because that has to be done, that also has to be done, but I sometimes had a question, and just waited.” (Quote #4, Geriatric patient, T1)

#### 3.2.2 Being unconscious incompetent

Nurses said that their mindset around movement during a hospital stay has changed positively, they were better able to work according to the principles of Function Focused Care in Hospital and experienced its added value.

“I have become more aware. I think that's very important. I was already doing it from time to time, applying FFC, actually, only now I am much more conscious of it.” (Quote #5, Geriatric nurse, T1)

“When I look from the beginning of the implementation of FFC, eh, in that respect I am also more aware, you know. That everything I do with my patients, with regard to basic care eh, that you have to do a bit extra to stimulate that self-reliance in the patient.” (Quote #6, Geriatric nurse, T2)

Patients said in the interviews that they know the importance of movement in general. They also said that they don’t recognize the principles of Function Focused Care in Hospital as a whole. Furthermore, patients stated that it depends on the individual nurse how they were stimulated and how movement was a topic that is talked about. Coaches also saw that nurses wanted to engage with the principles of Function Focused Care in Hospital, but did not see it in daily care.

“Well I think I don't get enough exercise. I almost dare not ask, because they are so busy, those girls. Uh, that care is so spread out, I hardly dare ask. But I should have more exercise, more walking, because I can do that now and I should actually, but I can't go out on my own very well yet, so… that's a bit difficult.” (Quote #7, Geriatric patient, T2)

“Um, well, look they could have… yes I don't know, I don't have to be in bed all the time now so they could have also said to me of well, you can, uh, walk to the coffee corner, or you can walk around once. That’s no problem. But I was never told.” (Quote #8, Neurology patient, T1)

The change in mindset of the nurses was measured with different questionnaires, the mean differences between the score before implementation and directly after implementation showed a change. All the following questionnaires showed positive changes in nurses: Barriers and Facilitators Assessment of Implementation (Δ 8.04, CI 2.58 – 13.49, p 0.01), Nurses Outcome expectations (Δ 2.17, CI 0.36–3.97, p 0.02), and the Self Efficacy Nurses (Δ 6.44, CI 2.52 – 10.37, p 0.00). Directly after implementation no significant differences were found for the knowledge test and the questions about the culture and environment. Although there were some differences directly after implementation, no significant differences were found for all questionnaires four months after implementation.

The perspective of the patients that it was dependent on individual nurses how movement was stimulated was confirmed by the patient file screening (*n* = 39). We observed that in 28.2 % of the screened files a short-term goal and in 25.6 % a long-term goal was set. In 7.7 % of the screened patient files a short-term goal was evaluated. Four months after implementation, nursing reports showed an increase of the reports about bathing (Δ9.4 %), dressing (Δ 10.7 %), mobility (Δ7.1), and a decrease of reports about the transfer (Δ - 2.0 %). The percentage of not reporting about these topics in the patient files were for washing 62.1 %, dressing 67.9 %, transfer 74.8 %, and mobility 58.8 %. The observations during ADL care show that before implementation some care activities are already according to the Function Focused Care in Hospital principles (*n* = 31, median 60 %, IQR 41 – 88 %). Directly after implementation, the adoption of Function Focused Care rises to a median of 83 % (*n*= 9, IQR 83 – 100 %), and four months after implementation drops to a median of 75 % (*n*= 32, IQR 57 – 89 %).

Therefore, the discrepancy between the nurses’ and patients’ experiences and observed adoption of the intervention indicated that nurses demonstrate unconscious incompetency in providing care according to the principles of Function Focused Care in Hospital.

#### 3.2.3 Need for nursing leadership and autonomy

Both the nurses and the coaches recognized the importance of adequately implementing Function Focused Care in Hospital, but felt that the team as a whole should stand for the intervention otherwise they expected that the application will fail. They felt that this could have been achieved by showing autonomy and nursing leadership for a change in care and innovation such as Function Focused Care in Hospital. They believed innovation and implementation is a team effort and cannot rely on individuals.

“I also think, The more colleagues start working with it, the more the topic comes alive and becomes part of your daily functioning.” (Quote #9, Neurology nurse, T2)

Coaches said in the focus group interview that they felt resistance from the team in the implementation of Function Focused Care in Hospital. They felt it was hard to convince the team and getting the team on board to apply the intervention as it was supposed to. They recognized that they needed to step and speak up.

“I think that we as a working group also fell short in that respect. We actually needed someone who could say: well, briefly; come on! We have to set goals.” (Quote #10, FFC Coach, T2)

Furthermore, nurses reported that they were often disturbed in what they consider essential tasks as nurses. This led to prioritizing tasks given by other disciplines over Function Focused Care in Hospital. While the collaboration with other disciplines was perceived as a strength by the nurses and coaches; other disciplines did not work according to the principles of Function Focused Care in Hospitals.

“Because I have the feeling, at least that is, maybe generalizing, but that the doctors at our ward are not eager to know what the patient is doing about bathing and so on. They just want to know can the patient go home.” (Quote #11, FFC Coach, T2)

Coaches felt initial resistance from other disciplines as it was experienced as taking over work that feel in their scope of practice. Nurses felt they needed to show more leadership by involving other disciplines in the importance and benefits of working with Function Focused Care in Hospital.

“In the beginning there was a lot of resistance from the physio and occupational and speech therapists, they really had the idea that we were in their way so there were also conversations […]. They just had the idea that we were taking things away from them when that was not the intention at all.” (Quote #12, FFC Coach T2)

#### 3.2.4 Added value to personalized care experiences

Nurses perceived Function Focused Care in Hospital as an approach that closely aligns with the core values of their profession (e.g., respect, compassion, and patient advocacy) and supported personalized care experiences by emphasizing the autonomy and active participation of patients and their family.

“Well, I think the patient's control is just great. And indeed, it's also something for nurses to be able to do something with, otherwise you're very quickly just implementing all kinds of medical policy, but we don't make all kinds of goals for ourselves from a nursing point of view.” (Quote #13, Neurology nurse, T2)

This alignment contributed to the delivery of Function Focused Care in Hospital. Indeed, nurses felt that Function Focused Care in Hospital encourages patients to take an active role in setting and working toward their individual physical activity goals, fostering a sense of control and engagement in their care.

“Yes a sense of self-worth eh. (…) most people are older and they are dependent and they often find that the worst thing there is. Being dependent on others. (…) older people like to be in control for as long as possible. Look, if you go against it, you get friction. If you encourage and they do well and you compliment them, sometimes you know, you see a grin on their face.” (Quote #14, Geriatric nurse, T2)

While patients recognized the importance of physical activity, this perspective typically predated their hospital stay, indicating that it is not merely a result of Function Focused Care in Hospital. Patients often felt more physically active at home, where they engaged in various domestic tasks and community activities, than during hospitalization, where they perceived themselves as passive care recipients. This perspective reinforced the expectation that nurses will take over tasks.

“No, but you do hear it a lot… I like watching those medical things. But um, yeah for me it's a pretty logical thing, I think um… if you're on bed for a long time then… those legs decrease in strength. Same if you've been in plaster, that leg. Then you only get a very thin leg too.” (Quote #15, Neurology patient, T1)

Furthermore, the hospital environment was often not experienced by nurses and patients as conducive to physical activity (e.g., limited space for walking in the wards), which could have hindered the implementation of Function Focused Care in Hospital. Additionally, unlike their home environment, the hospital setting felt often unfamiliar to patients and not tailored to their personal needs and preferences regarding physical activity. Patients stated that this lack of adaptation restricts physical activity during hospitalization and undermines the personalized care experiences that Function Focused Care in Hospital aims to promote.

“I know myself that I, um… That I walk more at home than here. Then I go to the toilet and then um, I walk to the kitchen and then I walk there. But here, you walk from the bed and then sit down here and you don't get any further.” (Quote #16, Geriatric patient, T2)

## 4 Discussion

The findings showed that the delivery of care according to the Function Focused Care in Hospital principles increased from 60 % in the control condition to 75 % in the intervention condition. Moreover, nurses said patients were only assisted when needed, and nurses asked about their abilities to perform ADL, encouraging them to do as much as possible. Nevertheless, patients did not recognize Function Focused Care and experienced differences among individual nurses regarding to movement. Observations in the patients’ files showed that nursing reports about washing, dressing and mobility slightly increased, but were still lacking in >50 % of the screened files. A core element of Function Focused Care in Hospital, the goal setting, was seen in a quarter of the screened files. The results suggest that the implementation fidelity was influenced by different working mechanisms and contextual factors which led to different experiences from nurses, coaches and patients in promoting patient engagement and functional independence. The synthesized findings showed the pressure on continuity of care, the observed phenomenon of being unconscious incompetent, the need for nursing leadership and autonomy, and the added value of Function Focused Care in Hospital to personalized care.

Nurses recognized the potential of Function Focused Care in Hospital to enhance continuity of care by setting goals that align the efforts of healthcare providers and actively involve patients in their care process. However, patients have noted a lack of continuity in the care they received, observing that nurses often work in their own distinct ways. Nurses identified the varying shifts and team compositions as threats to the continuity of care. An observational study showed that patients in Dutch hospitals typically have contact with the same nurse in only one or two shifts during their hospital stay (van Erp et al., 2024). Furthermore, research shows that a lack of continuity of care influences patient satisfaction (Saultz and Albedaiwi, 2004; Adler et al., 2010). Ideally, patients want to interact with only a few nurses during their hospital stay, fostering stronger relationships and ensuring more personalized care (Saultz and Albedaiwi, 2004; Adler et al., 2010). However, due to nursing staff shortages and the necessity of rotating shifts, this level of continuity is often unattainable. Our findings show that goal setting, reporting, and evaluating these goals might enhance continuity by ensuring that all nurses work towards the same objectives. The goal setting within Function Focused Care in Hospital not only aligns the efforts of the nursing team but also makes these goals clear to the patients, thereby improving their overall care experience.

We also observed that nurses often assess their actions differently compared to patients and coaches who view nurses’ performance from an external perspective. Over time, nurses say that the application of Function Focused Care in Hospital increased, but this increase is not recognized by patients and coaches. This discrepancy implies an overestimation of nurses about the application of Function Focused Care in Hospital. This overestimation is also described in literature, where individuals tend to overestimate their performance and find it challenging to recognize their limitations (Ehrlinger, 2008; Kruger and Dunning, 1999). This overestimation happens because those lacking skills in these areas must also gain the metacognitive ability to recognize their incompetence (Ehrlinger, 2008; Kruger and Dunning, 1999). This phenomenon is described in the hierarchy of competence as being unconscious incompetent (Keeley, 2021). As nurses become more skilled, they gain a better understanding of their own strengths and weaknesses (Keeley, 2021; Kruger and Dunning, 1999). One part of Function Focused Care in Hospital is the ‘motivation and mentoring’ component, in which the coaches mentor nurses to become better skilled in applying the intervention. The coaches involved in our study recognized that their role could have been more optimal. Improving their role with a focus on enhancing the skills of the nurses can lead to better implementation and application of Function Focused Care in Hospital. Research confirms that a form of self-reflection and seeking feedback can significantly enhance self-awareness and improve patient care (Posluns and Gall, 2020). It is also shown that coaches can promote behavioural implementation through skill building and peer mentorship (Morena et al., 2022). This implies that structured mentoring and continuous professional development are crucial for developing the coaches' role, overcoming the unaware-unskilled phenomenon, and achieving better patient outcomes.

Furthermore, our study reveals that nurses found it challenging to prioritize tasks within their profession, indicating a need for more autonomy to act according to their professional judgment. Another study also observes this phenomenon, which highlights that nurses often experience limited autonomy due to organizational constraints and role ambiguity (Oshodi et al., 2019). In this study, nurses struggled to integrate Function Focused Care in Hospital into the multidisciplinary team, indicating a need to strengthen nursing leadership. Effective collaboration within the multidisciplinary team requires clear communication, role clarity, and shared leadership among healthcare professionals (Verspuy and Van Bogaert, 2018; Lutfiyya et al., 2019). Improving nursing leadership during the implementation of interventions can be addressed through structured leadership training programs, mentorship, and fostering a culture of shared decision-making. Such approaches can empower nurses to take on leadership roles and effectively advocate for the integration of new care models within the multidisciplinary team (Moss et al., 2016). Furthermore, a supportive work environment fosters a sense of ownership, boosts confidence and motivation, and enhances clinical nursing leadership skills (Duprez et al., 2024). Therefore, to successfully implement Function Focused Care in Hospital, it is crucial to enhance nursing leadership and autonomy within the multidisciplinary team.

The focus on physical activity within the implementation of Function Focused Care in Hospital can be improved, as patients in this study experienced being more physically active at home compared to the hospital setting. A study by Brown et al. (2009) found similar results, showing that hospitalized patients often have significantly lower mobility levels than when they are at home (Brown et al., 2009; van Delft et al., 2019). Notably, the hospital environment hinders their mobility, advocating for a more movement-friendly hospital setting. Research on aligning the hospital environment with patients’ needs for physical activity suggests that minor, practical adjustments tailored to the patient can enhance mobility and overall patient satisfaction (Geelen et al., 2021). These insights highlight the need for hospitals to create environments that encourage physical activity, which can be achieved through targeted interventions and environmental modifications.

This process evaluation, alongside the effectiveness study of Function Focused Care in Hospital, showed that the positive effects regarding length of stay (decrease of 3.3 days) and patients were more often discharged to their home setting (Kok et al., 2024) were a result of the change in mindset of the nurses and the change in the provided care. Nevertheless, we showed potential improvements to potentially enhance the effectiveness by optimizing the implementation of Function Focused Care in Hospital.

## 5 Strengths and limitations

This study has several strengths. By incorporating both nurse and patient perspectives, we captured a more holistic understanding of implementing Function Focused Care in Hospital. We also measured different potential working mechanisms of the intervention. Research emphasizes the importance of integrating multiple perspectives in mixed-methods research to comprehensively understand complex issues (Creswell and Piano Clark, 2007). We repeatedly interviewed patients and nurses over time, which allowed us to monitor changes in their mindsets and experiences. Moreover, the inclusion of multiple hospital wards allowed us to evaluate the implementation of the intervention across different nursing teams and patient populations, increasing the generalizability of our findings. Researcher triangulation further strengthened this study by reducing individual researcher biases, thereby increasing the validity and reliability of the conclusions drawn from the data (Denzin, 2012).

However, there are also some limitations to this study that need further reflection. Pragmatic choices in data collection, such as limited patient file screening, led to quantitative data constraints. Nevertheless, the use of source triangulation provided a comprehensive view of the data, aligning with the principles of mixed-methods research (Clark et al., 2008). Unfortunately, we had a low response rate for some questionnaires. Furthermore, not all questionnaires were validated. Devellis (2021) emphasizes the importance of using well-designed questionnaires that are clear and relevant to the research objectives, even if they are not formally validated, as long as they have been used effectively in previous studies and are deemed suitable by experts (DeVellis and Thorpe, 2021). We selected those questionnaires that were clear and relevant for capturing changes in respondents’ views and behaviors. These questionnaires had also been used in previous studies on Function Focused Care in Hospital and were deemed suitable based on expert experience.

## 6 Conclusions

This study shows that Function Focused Care in Hospital is an approach that generally aligns with current practice. To optimize implementation and to empower nurses who work with the intervention, it is necessary that the contribution to the continuity of care is emphasized, nursing leadership and autonomy and the skills of the nurses are improved, and there is attention to the time constraints nurses experience to provide personalized care.

## Declaration of generative AI and AI-assisted technologies in the writing process

During the preparation of this work the author(s) used Grammarly and Microsoft Copilot in order to improve readability and language of the manuscript. After using this tool, the author(s) reviewed and edited the content as needed and take(s) full responsibility for the content of the published article.

## CRediT authorship contribution statement

**Selma Kok:** Writing – review & editing, Writing – original draft, Visualization, Validation, Software, Project administration, Methodology, Investigation, Formal analysis, Conceptualization. **Elke G.E. Mathijssen:** Writing – review & editing, Validation, Formal analysis. **Lisette Schoonhoven:** Writing – review & editing, Writing – original draft, Visualization, Supervision. **Carolien Verstraten:** Writing – review & editing, Data curation, Conceptualization. **Silke F. Metzelthin:** Writing – review & editing, Funding acquisition, Conceptualization. **Nienke Bleijenberg:** Writing – review & editing, Visualization, Supervision. **Janneke M. de Man-van Ginkel:** Writing – review & editing, Visualization, Supervision, Project administration, Methodology, Investigation, Funding acquisition, Formal analysis, Data curation, Conceptualization.

## Declaration of competing interest

The authors declare that they have no known competing financial interests or personal relationships that could have appeared to influence the work reported in this paper.
